# Effect of increased oxygen tension on flicker-induced vasodilatation in the human retina

**DOI:** 10.1038/jcbfm.2014.161

**Published:** 2014-09-24

**Authors:** Stefan Palkovits, Reinhard Told, Agnes Boltz, Doreen Schmidl, Alina Popa Cherecheanu, Leopold Schmetterer, Gerhard Garhöfer

**Affiliations:** 1 Department of Clinical Pharmacology, Medical University of Vienna, Vienna, Austria; 2 Center of Medical Physics and Biomedical Engineering, Medical University of Vienna, Vienna, Austria; 3 Department of Ophthalmology, Emergency University Hospital, Bucharest, Romania

**Keywords:** hemodynamics, neurovascular coupling, physiology

## Abstract

In the retina, blood flow and neural activity are tightly coupled. Stimulation of the retina with flickering light is accompanied by an increase in blood flow. The current study seeks to investigate whether an increase in oxygen tension modulates flicker (FL)-induced vasodilatation in the human retina. A total of 52 healthy volunteers were included. Via a breathing mask, 100% oxygen (O_2_) was administered in one, a mixture of 8% carbon dioxide and 92% oxygen (C/O) in a second cohort. Retinal vessel diameters were measured with a Vessel Analyzer and FL responses were assessed before and during the breathing periods. At baseline, FL stimulation increased retinal vessel diameters by +3.7±2.3% in arteries and by +5.1±3.7% in veins. Breathing of C/O led to a decrease in arterial (−9.0±6.9%) and venous (−11.3±5.9%) vessel calibers. Flicker response was increased to 5.7±2.5% in arteries and to 8.6±4.1% in veins. Breathing of pure O_2_ induced a vasoconstriction of vessel diameters by −14.0±5.3% in arteries and −18.4±7.0% in veins and increased FL responses in arteries (+6.2±2.8%) and veins (+7.2±3.1%). Systemic hyperoxia increases FL-induced retinal vasodilatation in the retina. The mechanism by which oxygen modulates the hyperemic response to FL stimulation remains to be elucidated.

## Introduction

Functional hyperemia, first described for the brain more than 100 years ago,^1^ is an important physiologic mechanism that allows for the adaptation of blood flow to changed metabolic demands of the tissue.^2^ As for the eye, it has been shown that an increase in retinal and optic nerve head blood flow can be evoked by stimulation with flickering light.^3,4^ The current view is that visual stimulation leads to augmented neural activity, which in turn increases the metabolic needs of the tissue and subsequently triggers the increase in blood flow.^5^ This functional hyperemic response, also termed as neuro-vascular coupling, is considered necessary for proper retinal function assuring sufficient supply with oxygen and nutrients.^2,6^ As such, it has been shown that several ocular diseases such as glaucoma^7^ or diabetic retinopathy^8,9^ are associated with impaired vasodilatation in response to flicker (FL) stimulation.

Although several different mediators such as nitric oxide, oxygen, glucose, or K^+^ have been proposed as potential mediators of the hyperemic response,^10^ the exact molecular mechanisms underlying FL-induced vasodilatation have not yet been identified.^2^ As for oxygen, *ex vivo* preparations indicate that O_2_ does not directly mediate vasodilatation but has a modulatory role on FL response.^11^ These observations, however, could not be confirmed in *in vivo* animal models, where an effect of exogenously administered O_2_ could not be shown.^11^ The authors ascribed this behavior to the pronounced vasoconstriction that occurs during 100% O_2_ breathing thereby regulating inner retinal oxygen tension. Whether increased oxygen delivery may alter neuro-vascular coupling in the human retina has never been tested experimentally.

The current study was designed to investigate the effect of increased oxygen partial pressure induced by pure oxygen breathing on FL-induced retinal vasodilatation. In addition, given that administration of pure oxygen leads to a pronounced vasoconstriction and decrease in blood flow, which might limit the enhanced oxygen delivery and the diffusion in the surrounding tissue of the vessel, we have also tested the effect of 8% carbon dioxide, a vasodilating agent, added to 92% oxygen on FL response. On the basis of previously published data, one can expect that the addition of carbon dioxide limits the vasoconstrictor effect of O_2_, which may allow for an even stronger oxygenation of the retinal tissue compared with pure oxygen alone.^12,13^

## Materials and methods

### Subjects

The study was conducted in adherence to the Good Clinical Practice guidelines and to the Declaration of Helsinki including all revisions. The study protocol was approved by the Ethics Committee of the Medical University of Vienna. Written informed consent was obtained from all subjects before inclusion. A total of 52 healthy, nonsmoking subjects were consecutively included in two different study cohorts if they met the inclusion criteria. All subjects had to pass a screening examination during the 4 weeks before the study day. The screening consisted of a physical examination including medical history, a blood draw to assess hematological status, urine analysis, and an ophthalmic examination. Subjects were only included if no ophthalmologic disease and no general disease were diagnosed. In addition, only subjects with ametropia less than three diopters were included.

### Description of Study Days

One study day was scheduled for each subject. Subjects had to abstain from caffeine or alcohol-containing beverages for at least 12 hours before the study day. After written informed consent was obtained, a total of 52 male and female subjects, aged between 18 and 35 years were included in one of two treatment groups. The first cohort received a gas mixture containing 8% carbon dioxide and 92% oxygen (C/O), whereas the second cohort received pure oxygen (O_2_).

In all subjects, one drop of tropicamid (Agepha, Vienna, Austria) was instilled before the measurements to induce pupil dilatation. After a resting period of at least 20 minutes to assure stable hemodynamic conditions, baseline (BL) measurements of retinal arterial and venous diameters were performed and the retinal FL response was determined. Oxygen partial pressure was measured using capillary blood drawn from the arterialized ear lobe. A first or second degree temporal artery and vein were selected for the vessel diameter measurements.

Thereafter, a 30-minute breathing period with either pure oxygen (O_2_) or the mixture containing 92% oxygen and 8% carbon dioxide (C/O) was scheduled. During the last 15 minutes of the breathing period retinal vessel diameters and FL response were reassessed and oxygen partial pressure measurements were repeated. During the whole study period systolic and diastolic blood pressure as well as heart rate and peripheral oxygen saturation were measured every 5 minutes for safety reasons.

### Measurement of Hemodynamic Parameters

Systolic, diastolic blood pressures and pulse rate were measured every 5 minutes on the upper arm by the Riva-Rocci method using a patient monitor (HP-CMS patient monitor, Hewlett Packard, Palo Alto, CA, USA).

### Measurement of Peripheral Oxygen Saturation

Oxygen partial pressure was determined with blood drawn out of the ear lobe after arterialization with nicotinate plus nonylvanillamid ointment (Finalgon, Boehringer Ingelheim Pharma GmbH & Co. KG, Ingelheim am Rhein, Germany). Blood was collected with a thin glass capillary, and pH, pCO_2_, pO_2_, and SaO_2_ were determined by an automatic blood gas analysis system.

### Measurement of Retinal Vessel Diameter

Retinal vessel diameters were assessed using a Dynamic Vessel Analyzer described previously.^14^ Briefly, the Dynamic Vessel Analyzer is a commercially available system (IMEDOS, Jena, Germany) for the accurate determination of retinal arterial and venous diameters. For this purpose, the fundus is imaged onto the charge-coupled device chip of the video camera. Due to the absorbing properties of hemoglobin each blood vessel has a specific transmittance profile. Measurement of retinal vessel diameters is based on adaptive algorithms using these specific profiles. In the present study, one major retinal temporal arteriole and veinule was selected for measurement. Measurements of retinal vessel diameters were taken between 1 and 2 disc diameters from the margin of the optic disc in one inferior temporal arteriole and one inferior temporal veinule. For evaluation of the FL response, 60 seconds of BL measurements of retinal vessel diameters were followed by 30 seconds of FL light stimulation. The stimulation frequency was 12.5 Hz.

### Statistical Analysis

All statistical analyses were done using the Statistica software package (Release 6.0, StatSoft Inc., Tulsa, OK, USA). Baseline characteristics of both groups were described using descriptive statistics. Shapiro–Wilk test was used to assure normal distribution of the data. Baseline values of vessel diameters were calculated as an average of the last 20 seconds before start of the light stimulation. Vessel diameters during FL stimulation were calculated as an average of the last 20 seconds of the stimulation period. Flicker-induced changes in retinal vessel diameters (retinal FL response) are expressed as percentage change over BL values, that is (FL−BL) × 100/BL. Two-tailed paired *t*-tests were used to determine statistical significance for dependent data. Unpaired *t*-tests were used to determine differences between groups. Bonferroni–Holm procedure for multiple testing was applied for both cohorts to reduce the type I error. All results are presented as means±standard deviation. A *P*<0.05 or smaller was considered as level of significance.

## Results

Baseline characteristics of both study groups are shown in Table 1. Systemic blood pressure was slightly higher in the O_2_ group than in the C/O group, all other BL parameters were comparable between groups. One subject of the C/O group had to be excluded from the analysis because of insufficient image quality of the measurements.

### Effect of 100% Oxygen (O_2_ Group)

**Figure 1:**
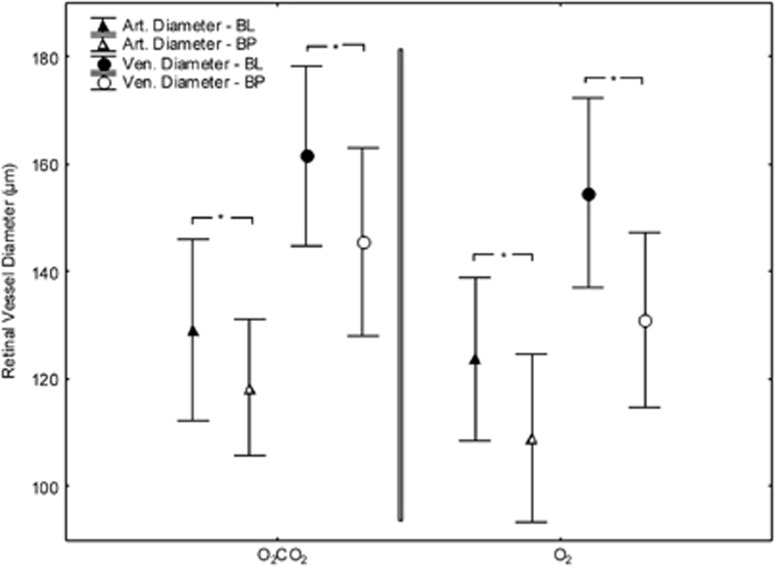
Retinal vessel diameters at BL and during inhalation of the O_2_/CO_2_ mixture (left) and pure O_2_ (right). Data presented as mean and standard deviation. Asterisks mark significant differences. BL, baseline; BP, breathing period.

**Figure 2:**
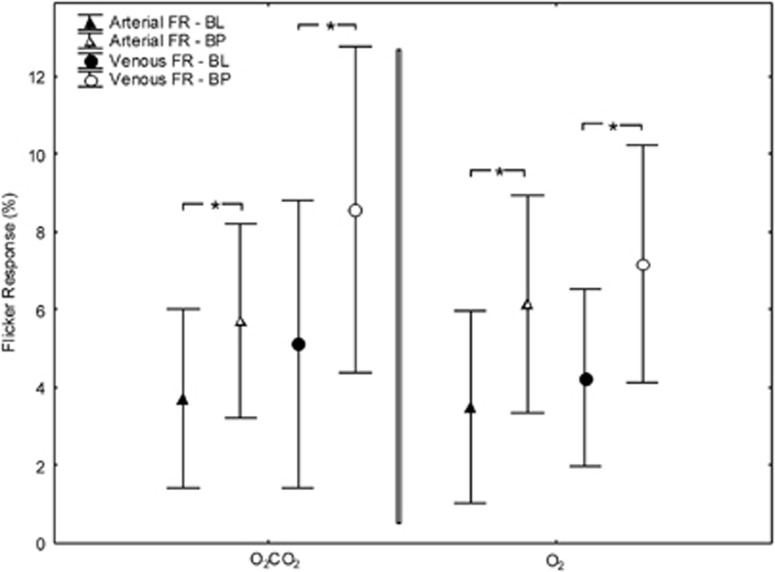
Flicker response (FR) at BL and during inhalation of the O_2_/CO_2_ mixture (left) and pure O_2_ (right). Data presented as mean and standard deviation. Asterisks mark significant differences. BL, baseline; BP, breathing period.

In the O_2_ group, FL-induced vasodilatation was 3.5±2.5% in retinal arteries and 4.2±2.3% in retinal veins at BL conditions (both *P*<0.01). Inhalation of pure oxygen increased pO_2_ from 88±8 mm Hg to 364±100 mm Hg (*P*<0.01). Likewise, oxygen saturation increased from 97.5±0.9% to 99.0±0.4% (*P*<0.01) during the O_2_ breathing period and pCO_2_ decreased from 37.5±3.7 mm Hg to 33.8±7.8 mm Hg (*P*<0.01). In addition, pure oxygen breathing induced a vasoconstriction of retinal vessel diameters by −14.0±5.3% (see Figure 1, *P*<0.01) in retinal arteries and by −18.4±7.0% in retinal veins (*P*<0.01). During inhalation of oxygen FL-evoked vasodilatation was significantly higher compared with BL conditions in both retinal arteries (+6.2±2.8%, *P*<0.01) and retinal veins (+7.2±3.1%, *P*<0.05) (see Figure 2).

### Effect of C/O

At BL conditions, FL stimulation increased retinal vessel diameters by +3.7±2.3% (*P*<0.01) in retinal arteries and by +5.1±3.7% (*P*<0.01) in retinal veins. Breathing of the C/O combination increased pO_2_ from 82±8 mm Hg to 274±70 mm Hg (*P*<0.01), pCO_2_ from 37.0± 3.0 mm Hg to 45.5±5.7 mm Hg (*P*<0.01) and blood oxygen saturation from 96±0.8% to 99±0.3% (*P*<0.01). The increase in pO_2_ was significantly higher in the O_2_ group than in the C/O group (*P*<0.01 between groups). The difference in the pCO_2_ response was also significant between the O_2_ group and the C/O group (*P*<0.01 between groups). Breathing of C/O induced a pronounced decrease in both retinal and venous vessel calibers, amounting to −9.0±6.9% (see Figure 1, *P*<0.01) in retinal arteries and −11.3±5.9% (*P*<0.01) in retinal veins. This decrease in vessel calibers was less pronounced during breathing of C/O compared with pure oxygen in both arteries (*P*<0.01) and veins (*P*<0.01). During C/O inhalation, FL-induced vasodilatation was significantly increased by 5.7±2.5% (*P*<0.05) in retinal arteries and by 8.6±4.1% (*P*<0.05) in retinal veins (see Figure 2). No significant difference in FL response during gas inhalation was observed between the O_2_ and the C/O group in retinal arteries (*P*=0.61) or retinal veins (*P*=0.24).

## Discussion

In this study, we tested the hypothesis that systemic hyperoxia as induced by inhalation of gases with high fraction of O_2_ may alter FL-induced vasodilation in the human retina. Our data indicate that under increased pO_2_, FL-induced vasodilation of retinal vessels is more pronounced when compared with normoxic conditions. To the best of our knowledge, this is the first study providing evidence that oxygen has a modulatory effect in FL-induced retinal vasodilatation in humans. Interestingly, this is in contrast to animal studies where systemic hyperoxia did not affect neurovascular coupling in the brain^15^ or the retina.^11^

The concept of neuro-vascular coupling assumes that a mechanism exists by which neurons can—either directly or indirectly—signal increased metabolic demands. Given that augmented neural activity results in increased oxygen consumption in the retina,^16,17^ it has originally been thought that a decrease in tissue oxygen may directly trigger vasodilatation. This hypothesis was especially tempting because it is known for a long time that oxygen is a potent regulator of cerebral and retinal blood flow. An increase in arterial pO_2_ as induced for example by oxygen breathing is accompanied by a pronounced vasoconstriction ^13^ and a decrease in retinal blood flow in humans^18^ and animal experiments.^19^ This reaction of the retinal vascular bed is crucial to keep local oxygen tension constant to avoid potentially toxic effect of high oxygen levels, such as the formation of free radicals.

Our results support the current view that a decrease in tissue oxygen levels does not directly trigger vasodilatation during photic stimulation of the human retina.^2,5,20^ Whereas it was originally hypothesized that a negative metabolic feedback mechanism is responsible for the hyperemic response to neuronal stimulation^2,5^ experimental evidence revealed that functional hyperemia can occur even without a decrease in local oxygen tension.^21,22^ The data of the current study show that under hyperoxic conditions, independently of the gas mixture used, FL responses are significantly increased compared with normoxic conditions. When interpreting these results, one has to consider that currently the exact extent of pO_2_ increase in the inner retinal during breathing gas mixtures with high oxygen contents is unkown. In animal models, the inner retinal tissue pO_2_ increases only slightly with breathing 100% oxygen^23, 24, 25^ with the oxygen gradient becoming steeper due to the diffusion of oxygen from the choroid to the retina.

This is compatible with recent human data indicating that oxygen extraction of the inner retina decreases by more than 60% during 100% oxygen breathing.^26^ Adding small amounts of CO_2_ to oxygen in the inhalate leads to a more pronounced increase of pO_2_ in inner retinal tissue by limiting the oxygen-induced vasoconstriction of the retinal vessels.^12^ This is also reflected in the results of the current study showing less retinal vasoconstriction in the C/O group as compared with the O_2_ group and is also keeping with our previous studies.^13^ As such we assume that in the present study exogenous O_2_ leads to a slight increase in local inner retinal tissue pO_2,_ which is more pronounced in the O_2_ group than in the CO_2_ group. If hypoxia would be the trigger of retinal vasodilation during FL stimulation then one would assume a reduced hyperemic response under such conditions. Hence, our data again support the view that neurovascular coupling is not directly related to a negative feedback mechanism with oxygen as a mediating signal.

Species differences in neurovascular coupling may well arise from anatomic differences. One has to consider that the circulatory systems considerably differ between animal models such as the rat retina compared with the human retina. As mentioned above in humans breathing of pure oxygen will lead to a fast and substantial increase in pO_2_ in the choroidal circulation, which is mainly a consequence of the lack of an oxygen-induced autoregulatory response of the choroidal circulation.^27^ This together with only smaller increased pO_2_ of the inner retina will considerably change the O_2_ gradient throughout the retina and, as a consequence, will lead to an increase in O_2_ diffusion to the inner layers of the retina. The exact effect of this substantial metabolic change in the retina is currently unknown, but may certainly affect neuro-vascular coupling.

*In vitro* studies in the brain and the eye show reduced neurovascular coupling when oxygen is added. Under such conditions there is a pronounced increase in local pO_2_ which appears to be more than 10-fold.^11^ Gordon *et al.*^28^ report that under such conditions in isolated brain slices hyperoxia reduces neuro-vascular coupling. In keeping with these results, experiments in isolated retinas show that hyperoxia reduces FL-induced vasodilatation.^11^

A strength of the present study is that it provides data from humans, which are obviously slightly different from those obtained in rats. This is, however, also a drawback of the study, because experiments that investigate the underlying mechanisms are limited due to ethical considerations. As such we can only speculate about the mechanisms underlying the augmentation of the retinal FL response. In the retinal *ex vivo* preparations, high levels of oxygen suppresses prostaglandin-mediated vasodilatation and HETE-20 mediated vasoconstriction after FL stimulation.^11^ The authors assumed that this may be related to the O_2_ dependence of the 20-HETE synthesis, which has a KmO_2_ of 60 to 70 mm Hg being considerably higher than the KmO_2_ for the COX-1 and COX-2 pathway. One potential explanation of the differences between humans and rats may also be related to the relative importance of the 20-HETE signaling in mediating neurovascular coupling. To the best of our knowledge, no human study has yet proven that there is an important HETE-20 mediated vasoconstrictor component to neurovascular coupling.

Another option would be that vasodilators producing enzymes with even higher oxygen dependence are involved in the process. A possible candidate could be neuronal nitric oxide synthase. It has been shown that the nitric oxide synthase-related nitric oxide production is largely coupled to the local O_2_ concentration^29^ and it appears to have a modulatory role in retinal neurovascular coupling in the retina.^30, 31, 32^ Obviously another hitherto unidentified vasodilator could have a role as well, but work has to continue in animal models to search for such a component.

Whereas several studies indicate that FL stimulation alters the oxygen consumption and pO_2_ especially in the inner retina in *in vivo* animal experiments,^17,33^ evidence for the human retina is relatively sparse. One of the few studies investigating oxygen saturation during visual stimulation reports increased venous oxygen saturation during flicker stimulation, whereas the oxygen saturation in retinal arteries remains stable.^34^ Whether this indicates that the FL-induced increase in blood flow overcomes the oxygen need from the tissue or is just appropriate to increase the intravascular pO_2_ sufficiently to establish the necessary pO_2_ gradient from the vessel to the surrounding tissue to maintain pO_2_ under increased oxygen consumption has yet to be clarified.

When interpreting our results one has also to consider several limitations of the current study. First and most importantly, the exact local pO_2_ in the different layers of the retina is not known. Although new technical developments allow now for the noninvasive measurement of oxygen saturation in selected vessels in humans,^35, 36, 37^ no technique is currently available to noninvasively assess pO_2_ in the retinal tissue.

Second, only major retinal arterioles and venules in the range of approximately 70  to 150 *μ*m have been measured. As the resistance to flow is mainly determined in the smaller arterioles and capillaries, data about vessel reaction in these vessels would be valuable. However, with the techniques currently available these small resistance vessels are not accessible for measurements.

Third, our data show that breathing of pure oxygen as well as the C/O mixture induces a pronounced vasoconstriction, which is in keeping with the results of previous studies in humans.^13^ Given that this strong vasoconstriction *per se* leads to changes in the vessel wall, such increased muscular tension of the smooth muscle cells in the vessel wall or an increased shear stress on the vascular endothelium, we cannot exclude that preconstricted vessels *per se* tend to dilate more pronounced compared with nonpreconstricted vessels.

Finally, BL systemic blood pressure was slightly higher in the O_2_ group than in the C/O group. Nevertheless, FL-induced retinal vasodilatation was not significantly different between groups making it unlikely that this imbalance influences our results to a major degree.

In conclusion, our results indicate that increasing systemic pO_2_ by breathing pure oxygen or a mixture of oxygen with carbon dioxide alters the hyperemic response of retinal vessels to stimulation with FL light. Although the exact reason for this altered FL response is unclear, our data support the hypothesis that neuro-vascular coupling in the retina is modulated by oxygen.

## Figures and Tables

**Table 1:** Baseline characteristics of both groups

	*O*_*2*_ *group (*n*=30)*	*C/O group (*n*=21)*	P
Sex (male/female)	16/14	11/10	
Age (years)	25±4	23±3	0.2
SBP (mm Hg)	116±11	110±10	<0.05
DBP (mm Hg)	72±8	61±7	<0.05
MAP (mm Hg)	89±8	78±7	<0.05
PR (beats per minute)	68±12	75±14	0.5
SpO_2_ (%)	98±1	98±2	0.5

DBP, diastolic blood pressure; MAP, mean arterial pressure; PR, pulse rate; SBP, systolic blood pressure; SpO_2_, peripheral oxygen saturation (pulse oxymetric module).
